# Human stem cell-based models for studying autism spectrum disorder-related neuronal dysfunction

**DOI:** 10.1186/s13229-020-00383-w

**Published:** 2020-12-11

**Authors:** Arquimedes Cheffer, Lea Jessica Flitsch, Tamara Krutenko, Pascal Röderer, Liubov Sokhranyaeva, Vira Iefremova, Mohamad Hajo, Michael Peitz, Martin Karl Schwarz, Oliver Brüstle

**Affiliations:** 1 Institute of Reconstructive Neurobiology, University of Bonn Medical Faculty & University Hospital Bonn, Venusberg-Campus 1, Building 76, 53127 Bonn, Germany; 2 Life & Brain GmbH, Platform Cellomics, Venusberg-Campus 1, Building 76, 53127 Bonn, Germany; 3 Institute of Experimental Epileptology and Cognition Research, University of Bonn Medical Faculty & University Hospital Bonn, Venusberg-Campus 1, Building 76, 53127 Bonn, Germany; 4 Cell Programming Core Facility, University of Bonn Medical Faculty, Bonn, Germany

**Keywords:** Autism spectrum disorder, Induced pluripotent stem cells, Cell reprogramming, Brain organoids, Neuronal connectivity, In vitro differentiation

## Abstract

The controlled differentiation of pluripotent stem cells (PSCs) into neurons and glia offers a unique opportunity to study early stages of human central nervous system development under controlled conditions in vitro. With the advent of cell reprogramming and the possibility to generate induced pluripotent stem cells (iPSCs) from any individual in a scalable manner, these studies can be extended to a disease- and patient-specific level. Autism spectrum disorder (ASD) is considered a neurodevelopmental disorder, with substantial evidence pointing to early alterations in neurogenesis and network formation as key pathogenic drivers. For that reason, ASD represents an ideal candidate for stem cell-based disease modeling. Here, we provide a concise review on recent advances in the field of human iPSC-based modeling of syndromic and non-syndromic forms of ASD, with a particular focus on studies addressing neuronal dysfunction and altered connectivity. We further discuss recent efforts to translate stem cell-based disease modeling to 3D via brain organoid and cell transplantation approaches, which enable the investigation of disease mechanisms in a tissue-like context. Finally, we describe advanced tools facilitating the assessment of altered neuronal function, comment on the relevance of iPSC-based models for the assessment of pharmaceutical therapies and outline potential future routes in stem cell-based ASD research.

## Introduction

According to the 5th edition of the ‘Diagnostic and Statistical Manual of Mental Disorders’, autism is a developmental disorder with impairments in social interaction and communication, which is characterized by restricted and repetitive behavior patterns [1]. Autism has an average worldwide prevalence of 0.6–1%. In about 5% of all cases, it is associated with syndromic forms of ASD such as fragile X syndrome (FXS; *FMR1* mutation), Rett syndrome (RTS; *MECP2* mutation), Angelman syndrome (15q11q13 deletion), Timothy syndrome (*CACNA1C* mutation), Phelan-McDermid syndrome (22q13 deletion) and Kleefstra syndrome (*EHMT1* mutation) [2]. Furthermore, there are several other genetic conditions, which are less stringently correlated to autism (reviewed in detail by [3]). Interestingly, genes related to synaptic transmission such as *NLGN3*, *NLGN4*, *NRXN1* and *SHANK3* have been shown to play an important role in the pathophysiology of non-syndromic forms of autism [4]. In addition, diverse environmental factors are considered to contribute to the development of the disease [5]. Since the clinical manifestations and pathological mechanisms of autism are highly heterogeneous, the disease is today referred to as autism spectrum disorder (ASD) (as is more comprehensively outlined in the review by [6]).

Despite the fact that different mechanisms might be involved in the pathogenesis of ASD, there is a general consensus and strong body of evidence that this disorder has a developmental origin. More than 30% of all pediatric ASD patients carry variants in genes that had previously been associated with developmental delay [7, 8]. Genetic studies in the 1990s revealed that genes affected in FXS and RTS (i.e., *FMR1* and *MECP2*) have important functions during nervous system development [9, 10]. For example, FMR1 controls the expression of the neural genes *SOX1* and *PAX6* and is considered highly relevant for neuronal differentiation [11]. In animal models of FXS, specifically fast-spiking GABAergic neurons are affected by a maturation deficit: Compared to control animals, *Fmr1* knockout (KO) mice exhibit GABAergic neurons with shorter and less branched dendrites, lower membrane capacitance and increased input resistance, alterations which indicate a delay in the functional maturation of this neuronal subtype [12]. Furthermore, hippocampal neurons of *Fmr1-*deficient mice show decreased expression of GABA receptor subunits, and the pharmacological blockade of mGluR5 is able to revert the animals’ learning and memory deficits as well as increased startle response [13, 14]. Concordant with this finding, *Fmr1* KO mice were reported to show an increase in the number of glutamatergic synapses in specific brain regions, such as the cortex and the hippocampus [15, 16].

In case of RTS, glutamatergic hyperfunction seems to play an important role, too, since treatment of mice deficient for the 5mC-binding transcription factor Mecp2 with the mGluR5 negative allosteric modulator CPET and the NMDAR antagonist ketamine reduces RTS-associated phenotypes such as deficits in cognition and information processing, respectively [17, 18]. Lack of Mecp2 was further found to interfere with the cell cycle dynamics of neural progenitor cells (NPCs) and their transition into more mature stages, as indicated by the delayed maturation of cortical neurons observed in Mecp2-deficient mice [19]. Moreover, *Mecp2* KO results in impaired dendritic arborization as well as deficiencies in synapse formation and network integration of newborn neurons in the adult mouse hippocampus [20].

Together, these finding suggest that alterations in glutamatergic and GABAergic neurons and thus excitation–inhibition (E/I) balance might be one of the key pathogenic mechanisms underlying ASD. In some ASD cases E/I imbalance might also result from impaired glutamatergic neurotransmission, since in a recent study loss-of-function mutations in the AMPAR gene *GRIA2* were found in 8 out of 28 patients diagnosed with ASD or ASD-related phenotypes [21]. Furthermore, mutations in *GRIA3* and NMDAR-coding genes (e.g., de novo mutations in *GRIN2A*, truncating mutations in *GRIN2C*, *GRIN3A* and *GRIN3B* and *GRIN2B* gain-of-function mutations) as well as alterations in metabotropic receptor subunits, especially *GRM1* and *GRM5*, have been linked to psychiatric and neurodevelopmental disorders including ASD [22].

Neuroimaging and *post mortem* studies provide additional evidence for altered neural connectivity in ASD patient brains, reinforcing the assumption that a neurodevelopmental component and especially an evolving E/I imbalance are integral parts of ASD pathogenesis. Structural magnetic resonance imaging (MRI) has revealed that in ASD, brain overgrowth during infancy is followed by a relative decrease in brain size of the frontal and temporal cortices during pre-adolescence and adulthood [23, 24], and functional MRI scans in adult ASD patients hint to neuronal hyperconnectivity between cortical and subcortical regions such as amygdala and thalamus [25]. *Post mortem* studies have revealed higher spine densities on glutamatergic cortical projection neurons of non-syndromic ASD patients [26]. Vice versa*,* patients with Angelman syndrome were reported to exhibit reduced cortical expression of GABA_A_ receptor [27]. Morin-Parent et al. recently found that FXS patients show reduced activity of GABAergic neurons, resulting in cortical hyperexcitability [28]. Interestingly, brain MRI scans of macaques carrying a mutation in *SHANK3* revealed region-specific hyper- as well as hypoconnectivities: While hyperconnectivity was evident in the somatosensory cortex, extrastriate cortical areas and the posterior cingulate cortex, other brain regions such as the medial prefrontal cortex, thalamus, striatum and motor regions were hypoconnected. These complex deviations in neuronal connectivity were associated with behavioral abnormalities characteristic for ASD in humans such as repetitive behaviors as well as motor, social and learning deficits [29].

Considering a neurodevelopmental origin of ASD pathogenesis, the aim of this review is to provide a concise summary of the recent advances and findings in the field of induced pluripotent stem cell (iPSC)-based modeling of ASD. Since iPSC models are widely used to mimic key aspects of early human (neuro)development, they are considered particularly useful for investigating ASD-related alterations in neuronal function and connectivity.

## Cell reprogramming and genome editing as basis for disease modeling

The iPSC technology provides a unique opportunity to reprogram somatic cells from healthy or diseased individuals to an embryonic stem cell (ESC)-like stage [30, 31]. Thus, iPSCs represent an expandable cellular resource for the derivation of a huge variety of somatic cell types, which can be used for developmental studies, disease modelling and pharmaceutical compound screening in vitro. In the context of brain research, human pluripotent stem cells (PSCs) have opened an alternative route to generate otherwise inaccessible patient-specific neural cells in virtually unlimited numbers in vitro. The validity and applicability of PSC-based models depend heavily on the quality of the PSC-derived neural cultures [32]. Most importantly, PSCs and their derivatives have to be rigidly quality controlled for genomic integrity, for instance by comparative genomic hybridization, single nucleotide polymorphism (SNP) analysis and prospectively also exome or whole genome sequencing [33]. In order to address topics such as E/I imbalance, neuronal differentiation of PSCs has to be highly standardized. For example, it has been shown that even very small fluctuations in the fraction of GABAergic neurons can have profound effects on the overall in vitro network activity [34].

Traditionally, extrinsic factor-guided protocols have been used to drive neuronal differentiation of PSCs. For the generation of cultures highly enriched for excitatory cortical projection neurons, the group of Frederick Livesey published a three-step protocol starting with the directed differentiation of PSCs into cortical precursors by combining dual SMAD inhibition [35] with activation of the retinoid signaling pathway [36]. These cortical precursors subsequently undergo functional maturation and form neuronal networks consisting of approximately equal proportions of upper and deep layer excitatory cortical neurons [36]. Further addition of the MEK/ERK inhibitor PD0325901 and the gamma-secretase/notch-signaling inhibitor DAPT results in almost pure neuronal cultures consisting of approximately 90% projection neurons staining positive for either the cortical layer V marker CTIP2 or the cortical layer VI marker TBR1 after 8 weeks of differentiation [37]. With this system, functional human autapses exhibiting electrophysiological properties comparable to those of primary mouse cortical neurons can be established [38]. In addition to the development of protocols generating excitatory cortical neurons, strategies for the differentiation of cortical inhibitory interneurons were published. In 2013, two independent groups revealed that activation of *FOXG1* and sonic hedgehog signaling via a combination of dual SMAD inhibition with the tankyrase inhibitor XAV939 and SHH induces a ventral telencephalic fate in ESCs resulting in efficient generation of medial ganglionic eminence-like precursors [39, 40]. After a 30-day long co-culture with primary mouse cortical pyramidal neurons [34] and glial cells [35], approximately 80% of the neurons derived by these protocols displayed characteristics of functional GABAergic neurons.

**Fig. 1:**
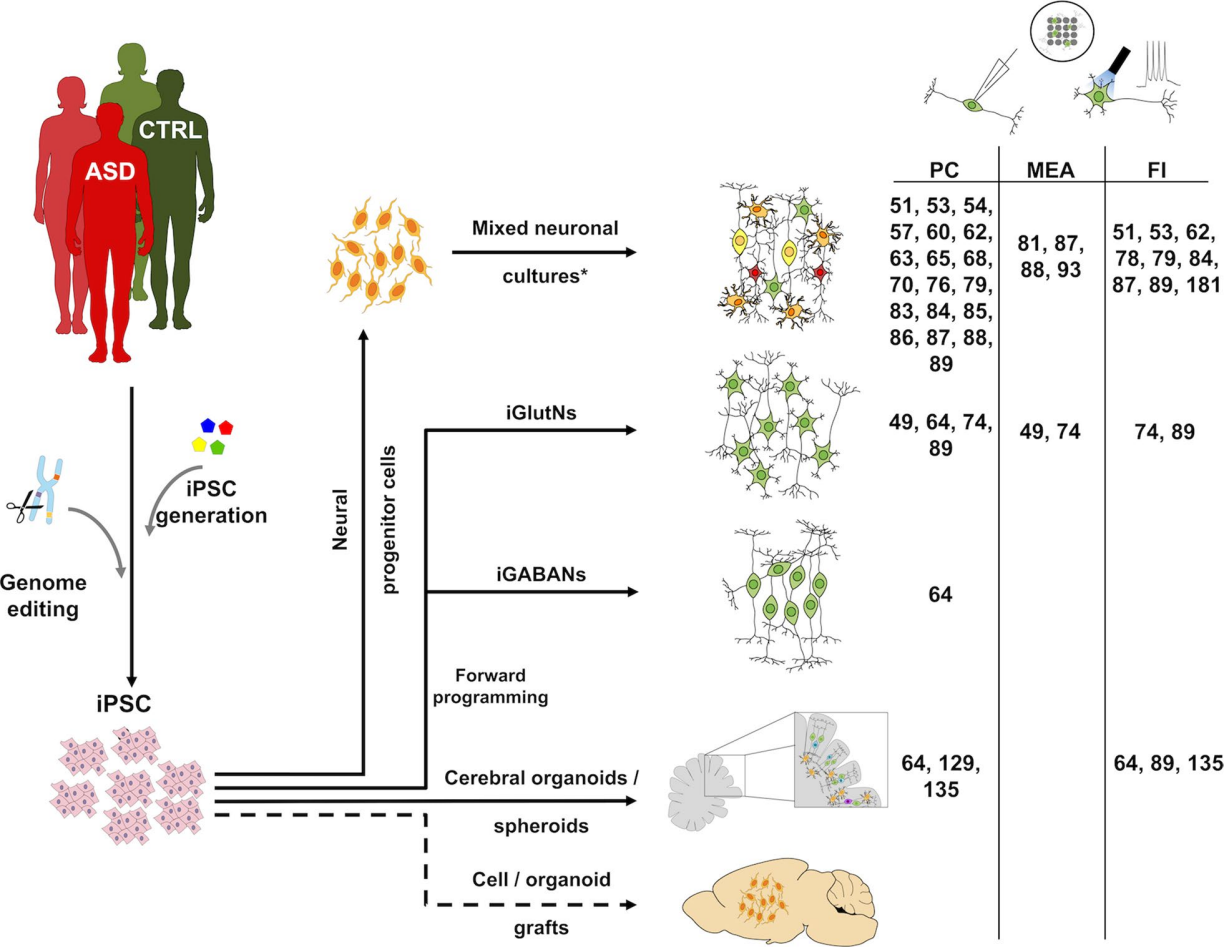
Cellular systems and functional read-outs used for stem cell-based modeling of ASD. Patient-specific and control iPSC lines are generated by classic reprogramming. Genome editing enables the repair of disease-related genetic variants or targeted insertion of ASD-related mutations into a control background, thereby providing isogenic pairs of disease-specific and control iPSC lines. Extrinsic factor-based differentiation is used to generate mixed neuronal cultures, which can be enriched for excitatory or inhibitory neurons depending on the culture conditions. More precise lineage specification can be achieved by transcription factor-based forward programming into induced glutamatergic or GABAergic neurons (iGlutNs or iGABANs; see also Table 1). 3D models such as cerebral organoids or xenotransplantation of human cells into the rodent brain might be used to study pathophenotypes in a tissue-like context. So far, ASD-related functional alterations have mainly been studied using patch clamping (PC), multi-electrode arrays (MEAs) and functional imaging (FI). Components of the figure were adapted from Servier Medical Art (https://smart.servier.com/#). CTRL: control; *Mixed neuronal cultures might be enriched for e.g. glutamatergic neurons

Although these protocols yield substantially enriched cultures of specific neuronal subtypes relevant for ASD and might even partially recapitulate in vivo neurodevelopment [36], they usually require extended periods of time to achieve cell fate specification and functional maturation, thereby limiting their applicability for standardized neuronal connectivity studies. For this purpose, other approaches such as forward programming, which refers to transcription factor-facilitated differentiation of PSCs towards a specific cell fate, might be of great use (Fig. 1). In 2013, the groups of Thomas Südhof and Marius Wernig reported a protocol for the rapid induction of functional cortical excitatory neurons from PSCs by lentiviral overexpression of the single transcription factor NGN2 [41]. A few years later, the same groups suggested a similar method for the generation of GABAergic neurons using transient overexpression of ASCL1 and DLX2 [42]. We employed genome editing to refine these protocols by expressing the transcription factors in an inducible manner from the human AAVS1 genomic safe harbor locus. This approach results in improved homogeneity and standardization of the induced neuronal cultures, yielding up to 90% of VGLUT2-positive and GABA-expressing neurons after overexpression of NGN2 or ASCL1 plus DLX2, respectively [38, 43].

The fact that PSCs are easily amenable to genome editing can be exploited for disease modeling by generating isogenic controls, either by repairing existing disease-causing mutations in patient lines or by newly introducing mutations in healthy control cell lines, mimicking genetic disease preconditions (Fig. 1). This strategy might be particularly relevant for modeling genetic variants with rather subtle effect size [44], since it isolates the effects of a specific mutation and is not biased by additional interindividual differences present in non-isogenic comparisons. Indeed, a number of mutations discussed in the context of ASD show low penetrance [45]. While the exact reasons for this phenomenon can be manifold, it is conceivable that in many cases low penetrance mutations elicit rather subtle changes in neuronal functioning, which become symptomatic only in combination with additional predisposing factors. Here, genome editing-based studies on an isogenic background can increase the resolution of subtle pathogenotypes, although modeling of low effect sizes in PSC-based systems remains a challenge.

Notably, CRISPR/Cas9-based genome editing has already been successfully used for modeling ASD. For example, a recent study addressed the role of the CREB pathway—which has been reported to directly regulate the expression of genes related to dendritic arborization [46] and indirectly influences MECP2 levels [47]—in RTS using a portfolio of ESC and iPSC lines comprising a patient-specific *MECP2* mutant line, a CRISPR/Cas9-corrected patient line and a control cell line in which *MECP2* was knocked out [48]. In another study by Deneault et al., CRISPR/Cas9 was used to establish 10 isogenic iPSC lines, each carrying KOs of different ASD-relevant genes, with the aim to reveal common disease-related phenotypes [49]. The results of this approach are depicted in more detail in the next paragraph. An additional asset of using genome-edited PSCs for disease modeling is the feasibility to mimic polygenic diseases by simultaneously inducing or repressing the expression of multiple disease-associated genes in one cell line. This can be achieved by the use of multiple guide RNAs directing complexes of inactive Cas9 mutants fused to transcriptional activators or repressors to their respective DNA target sites. Schrode et al. used this approach to simultaneously modulate the expression of 4 different genes (*FURIN*, *SNAP9*1, *TSNARE1* and *CLCN3*), for which SNPs have been reported to be related to schizophrenia (SCZ) [50]. Thus, this concept might also be informative for investigating non-syndromic forms of ASD or for probing low penetrance mutations in a defined genetic background harboring already other disease-related variants.

## ASD-related phenotypes in 2D models

In general, alterations in neuronal differentiation and maturation have the potential to elicit functional consequences. Indeed, human neurons derived from patient ASD iPSCs or PSCs engineered to carry ASD-related genetic variants have been reported to display a number of changes in their intrinsic properties, which can be expected to alter connectivity and network activity (Table 1).

**Table 1:** Cell biological and functional phenotypes revealed in 2D and 3D PSC-derived models of ASD

Genetic variant (syndrome)	Donor source	Differentiation approach	Neural derivative	Key phenotypic changes	References
*FMR1* (Fragile X syndrome)	ESC	EF	Neurons (generic)	Decreased rise time and increased duration of action potentials and spontaneous synaptic currents Decreased frequency of action potential firing Lack of responsiveness to glutamate	[11]
	iPSC		NPCs Neurons (generic)	Decreased neurite length Augmented intracellular Ca^2+^ responses to AMPA and kainate mediated by Ca^2+^-permeable AMPARs that lack the GLUA2 subunit Increased proportion of Ca^2+^-permeable AMPAR and NMDAR-co-expressing progenitors Increased expression of miR-181a, which represses translation of *GLUA2* transcripts	[51]
			Neurons (generic)	Increased expression of *REST* Several miRNAs dysregulated including hsa-miR-382 Downregulation of genes involved in axon pathfinding such as *ROBO3*, *DCC* and *SLIT1*	[52]
*MECP2 (Rett syndrome)*	ESC and iPSC	EF	Neurons (generic)	Decreased neurite outgrowth and dendritic arborization	[48]
	iPSC		Neurons (mixed)	Decreased neuronal soma size Decreased spine densities Decreased number of glutamatergic synapses (partially rescued by overexpression of MECP2 or IGF1 treatment) Decreased frequency of activity-dependent Ca^2+^ oscillations Decreased EPSC frequency	[53]
			Neurons (generic)	Decreased expression of KCC2 leading to a delayed GABA functional switch from excitation to inhibition	[54]
			Neurons (mixed)	Decreased neuronal soma size Decreased dendritic arborization Increased input resistance Decreased membrane capacitance Decreased action potential frequency	[57]
	iPSC			Decreased astrogenesis	[91]
	iPSC and ESC		Neurons (GABAergic) Astrocytes	Decreased neurite length and decreased number of terminal ends of wild-type interneurons after co-culture with mutant astrocytes Increased neurite length and increased number of terminal ends of mutant interneurons after co-culture with wild-type astrocytes	[92]
	iPSC		Organoids	Decreased thickness of the ventricular wall but increased ventricular areas Increased number of NPCs Reduced expression of neuronal markers	[134]
*CDKL5*	iPSC	EF	Neurons (generic)	Decreased number of excitatory synapses with aberrant spine structure	[60]
*15q11-q13: UBE3A (Angelman syndrome)*	iPSC	EF	Neurons (mixed)	Impaired maturation of resting membrane potential Decreased action potential frequency Decreased spontaneous EPSC frequency and synaptic plasticity Decreased frequency of Ca^2+^ transients	[62]
	ESC, iPSC	FP	Neurons (glutamatergic, GABAergic)	Increased action potential frequency Increased amplitude of the fast components after hyperpolarization	[64]
	ESC	EF	Organoids	Increased action potential frequency Increased frequency and amplitude of Ca^2+^ transients	
*15q11-q13 duplication syndrome*	iPSC	EF	Neurons (mixed)	Delayed action potential maturation Increased frequency of spontaneous action potential firing Increased synaptic event frequency and amplitude Impaired activity-dependent synaptic plasticity and homeostatic synaptic scaling	[63]
*CACNA1C* (Timothy syndrome)	iPSC	EF	Neurons (mixed)	Defects in intracellular Ca^2+^ signaling and activity-dependent gene expression Decreased fraction of neurons expressing lower cortical layer markers Abnormal expression of tyrosine hydroxylase Increased production of norepinephrine and dopamine Defects in action potential firing	[65]
			Spheroids	Increased Ca^2+^ levels following electrical depolarization Delayed interneuron migration (rescued by pharmacological modulation of L-type calcium channels)	[135]
*SHANK3 (Phelan-McDermid syndrome)*	iPSC	EF	Neurons (mixed)	Decreased neuronal soma size Decreased growth cone area, neurite length and neurite branching Dysregulation of genes associated with neuron projection, motility and regulation of neurogenesis Defects in excitatory and inhibitory synaptic transmission	[68]
	ESC	FP	Neurons (glutamatergic)	Decreased neuronal soma size Decreased neurite length and branching Impaired I_h_-channel function leading to increased neuronal input resistance and enhanced neuronal excitability Decreased EPSC frequency and amplitude	[69]
	iPSC	EF	Neurons (glutamatergic)	Decreased dendritic spine densities as well as whole spine and spine head volumes	[70]
	iPSC and ESC		Placodal neurons	Decreased neuronal soma size Increased neurite branching Decreased motility Decreased number of synaptic puncta	[71]
	iPSC		Neurons (glutamatergic)	Decreased number of synaptic puncta (rescued by IGF1 treatment) Increased input resistance Decreased amplitude and frequency of AMPA- and NMDA-EPSCs (rescued by SHANK3 expression)	[73]
	iPSC and ESC			Decreased intensities and frequencies of spontaneous Ca^2+^ oscillations (rescued by lithium and VPA treatment)	[181]
*EHMT1* (Kleefstra syndrome)	iPSC	FP	Neurons (glutamatergic)	Upregulation of *GRIN1*, encoding the NMDAR subunit 1 Decreased burst frequency and prolonged inter-burst interval (rescued by chronical treatment with the NMDAR channel pore blocker MK-801) Increased burst duration (rescued by chronical treatment with MK-801) Divergent spike organization with fewer spikes occurring outside network bursts Irregular network bursting (rescued by chronical treatment with MK-801)	[74]
*SHANK2*	iPSC	EF	Neurons (glutamatergic)	Increased dendrite length Increased number of synapses Upregulation of genes associated with neurodevelopment Compromised activity-dependent dendrite extension Increased EPSC frequency	[76]
*NLGN4* (R704C variant)	ESC	FP	Neurons (glutamatergic, GABAergic)	Increased number of excitatory synapses Increased miniature EPSC frequency	[77]
*NRXN1*	iPSC	EF	Neurons (glutamatergic)	Increased frequency, duration and amplitude of Ca^2+^ transients Dysregulation of genes associated with ion transport and transporter activity	[78]
	iPSC		NPCs Neurons (mixed)	Decreased NPC proliferation Increased astrogenesis Decreased amplitude and slower rise time of Ca^2+^ transients Decreased action potential amplitude	[79]
	ESC		Neurons (glutamatergic)	Increased levels of the critical synaptic scaffolding protein CASK Decreased miniature EPSC frequency	[80]
	iPSC		Neurons (mixed)	Decreased neuronal network activity	[81]
	iPSC and ESC		Neurons (generic)	Impaired astrocyte differentiation	[90]
*16p11.2 CNV*	iPSC	EF	Neurons (generic)	*Deletion* Increased neuronal soma size Increased dendrite length and neurite branching Decreased number of synapses Decreased neuronal excitability *Duplication* Decreased neuronal soma size Decreased dendrite length and neurite branching Decreased number of synapses Increased neuronal excitability	[83]
*TRPC6*	iPSC	EF	NPCs Neurons (mixed)	Decreased neurite length (rescued by treatment with the TRPC6-specific agonist hyperforin and overexpression of TRPC6) Decreased neurite branching (rescued by treatment with hyperforin and overexpression of TRPC6) Decreased density of dendritic spines (rescued by treatment with hyperforin and overexpression of TRPC6) Decreased density of VGLUT1 puncta (rescued by treatment with hyperforin and IGF1 as well as overexpression of TRPC6) Decreased Ca^2+^ influx in NPCs Decreased Na^+^ current densities	[84]
*lncRNA PTCHD1-AS*	iPSC	EF	Neurons (generic)	Decreased frequency of AMPAR-mediated miniature EPSCs Decreased NMDA-evoked current amplitude	[85]
*AFF2/FMR2 ANOS1 ASTN2* *ATRX CACNA1C* *CHD8 DLGAP2* *KCNQ2 SCN2A* *TENM1*	iPSC (isogenic)	FP	Neurons (glutamatergic)	Decreased capacitance: *ATRX-*, *SCN2A-*null neurons Increased action potential threshold: *ATRX-*, *CHD8-*, *SCN2A-*, *TENM1-*null neurons Decreased action potential amplitude: *SCN2A-*null neurons Decreased spontaneous EPSC frequency: *AFF2*-, *ASTN2*-, *ATRX*-, *KCNQ2*- and *SCN2A*-null neurons Decreased mean firing rate and network burst frequency: *SCN2A*-null neurons	[49]
*SETD5* *ERP44* *SKIV2L*	iPSC	EF	Neurons (generic) Astrocytes	Decreased number of synapses Decreased glutamate release Decreased spontaneous neuronal spiking activity	[93]
Unknown	iPSC	EF	Neurons (mixed)	Altered expression of 161 genes of which 22 have previously been associated to ASD Decreased Na^+^ and fast inactivating K^+^ voltage-gated currents Earlier action potential saturation Decreased spontaneous EPSC frequency and half width	[86]
			Neurons (glutamatergic)	Decreased Ca^2+^ transients Decreased spontaneous neuronal spiking	[87]
			NPCs Neurons (mixed)	Increased NPC proliferation correlating with the patients’ brain volumes (rescued by lithium chloride treatment) Increased number of inhibitory precursors and neurons (further facilitated by IGF1 treatment) Decreased number of glutamatergic precursors and neurons Decreased excitatory synapse density Decreased number of network bursts (rescued by IGF1 treatment)	[88]
		EF and FP	Neurons (generic, EF) Neurons (glutamatergic, FP) Organoids	Increased neurite length and branching observed in neurons derived in 2D by EF and in 3D organoids	[89]
		EF	Organoids	Upregulation of *FOXG1* Altered expression of genes related to cell proliferation, differentiation and synaptogenesis Differentiation bias towards GABAergic phenotype (rescued by shRNA-mediated knockdown of FOXG1)	[129]
*CHD8*	iPSC	EF	Organoids	Upregulation of *DLX* genes associated with GABAergic interneuron differentiation	[130]
			NPCs Neurons (mixed)	Dysregulation of genes implicated in neural development	[131]
			NPCs	Downregulation of genes implicated in neuronal differentiation	[132]
*RAB39B*	iPSC	EF	Organoids	Increased NPC proliferation Impaired differentiation of NPCs	[133]

Neurons (generic): no subtype characterization provided; neurons (mixed): population includes multiple subtypes; neurons (glutamatergic): population predominantly consisting of glutamatergic neurons

*EF* extrinsic factor-guided differentiation, *FP* forward programming

### Functional phenotypes detected in iPSC-based models of FXS and RTS

An early study based on FXS ESCs revealed that the disease-associated lack of *FMR1* expression leads to the derivation of NPCs with impaired neuronal maturation and increased gliogenic potential. Although FXS neurons were able to generate action potentials, they showed little spontaneous synaptic activity and no responsiveness to glutamate [11]. Achuta et al. identified various functional alterations in FXS iPSC-derived neural progenitors, including an increased differentiation of cells expressing AMPA receptors. The cells exhibited augmented intracellular calcium responses to AMPA and kainate that were mediated through calcium-permeable AMPARs lacking the GLUA2 subunit, which is encoded by *GRIA2*. The authors discuss that this could be due to a scenario where the loss of the RNA-binding protein FMRP leads to increased levels or mislocalization of the *GRIA2*-repressive microRNA miR-181a [51]. In addition, FXS iPSC-derived neurons generated in another study were found to show hsa-miR-382-dependent elevated *REST* levels leading to a downregulation of genes involved in axon pathfinding such as *ROBO3*, *DCC* and *SLIT1* [52].

RTS patient iPSC-derived neurons, too, exhibit morphological abnormalities such as a reduced soma size and decreased synaptic density, as well as functional deficits including altered calcium signaling, a decreased frequency of excitatory postsynaptic currents (EPSCs) [53] and a delayed GABA functional switch from excitation to inhibition, which is due to decreased expression of the MECP2 target gene *KCC2* [54]. An iPSC-based model of RTS was further used to analyze the potency of selected compounds to revert the mutation-associated reduction in MECP2 protein levels. More specifically, the authors tested for the effect of low dosages of gentamicin and treatment with IGF1 [53]. In prokaryotes, the aminoglycoside gentamicin possesses the ability to suppress premature stop mutations by binding 16S rRNA, thus impairing ribosomal proofreading. In eukaryotic cells, gentamicin was shown to induce low levels of translational misreading, too [55]. In human RTS-derived neuronal cultures, gentamicin treatment increased MECP2 protein levels and rescued the number of glutamatergic synapses [53]. IGF1 treatment was reported to partially alleviate RTS-like symptoms in Mecp2-deficient mice [56] and resulted in increased numbers of glutamatergic synapses on RTS patient-derived neurons, too [53]. Interestingly, even mutations affecting only one of the two main MECP2 isoforms are sufficient to provoke RTS-associated phenotypes: MECP2e1 isoform-deficient patient iPSC-derived neurons were reported to show reduced soma size, less complex dendritic arborization, increased input resistance, reduced membrane capacitance and lower frequency of action potential firing [57]. In contrast to RTS iPSC models, which are associated with a downregulation of MECP2, an increased number of synaptic puncta and synchronous bursting activity were detected in a model of *MECP2* duplication [58]. IPSC-derived neurons from patients with mutations in *CDKL5*, which typically present with a clinical phenotype similar to RTS [59], also display a reduced number of excitatory synapses with aberrant spine structure [60]. It remains to be investigated, however, whether these changes result from similar pathogenic mechanisms.

### Neuronal dysfunction in stem cell models of other syndromic forms of ASD

Angelman syndrome, which is caused by a deletion of the maternal 15q11-q13 chromosomal region, results in a complete lack of *UBE3A* expression, since the paternal allele encoding for this ubiquitin protein ligase is normally not expressed due to genomic imprinting [61]. In 2017, Fink et al. demonstrated that neurons differentiated from Angelman syndrome patient iPSCs via an extrinsic factor-guided neuronal differentiation protocol exhibit impaired neuronal maturation, as characterized by a more depolarized resting membrane potential and a reduced proportion of neurons firing mature action potentials, as well as decreased excitatory synaptic activity and a lower capacity for activity-dependent synaptic plasticity [62]. Vice versa, neurons differentiated from iPSCs of patients with duplication of the Angelman syndrome-affected chromosomal region exhibited higher EPSC amplitudes and frequencies [63]. In another recent study, the consequences of *UBE3A* mutations were assessed using NGN2-induced patient neurons and brain organoids. Here, the authors found that calcium- and voltage-dependent big potassium (BK) channels undergo UBE3A-mediated ubiquitination and subsequent proteasomal degradation. Consequently, loss of UBE3A leads to an increased activity of BK channels, which in turn causes neuronal hyperexcitability along with network synchronization. Indeed, antagonists for the affected BK channel could normalize neuronal excitability, pointing to BK channels as potential drug targets for the treatment of Angelman syndrome patients [64].

Despite the many advantages of protocols that are highly tailored towards the generation of a specific PSC-derived neuronal subtype, it might be still informative to use less stringent neuronal differentiation methods, since divergent in vitro neuronal subtype specification might be itself a disease phenotype. Paşca et al., for example, found that neuronal cultures generated from Timothy syndrome iPSCs—carrying mutations in *CACNA1C*, which encodes for the L-type calcium channel Ca_v_1.2—exhibit a sustained rise in intracellular calcium after depolarization and an increase in action potential width. Resulting activity-dependent gene expression changes were enriched for genes related to calcium-dependent CREB signaling, including the CREB downstream target *TH*. The authors further revealed that although neuronal differentiation of Timothy syndrome iPSCs mainly gave rise to neurons with cortical identity, these cells showed abnormally high expression levels of tyrosine hydroxylase and secreted more catecholamines such as dopamine and norepinephrine than control neurons. Treating patient iPSC-derived neurons with the L-type calcium channel modulator roscovitine decreased the number of tyrosine hydroxylase-positive neurons [65].

Another well-established genetic variant for ASD is SHANK3 deficiency, which is associated with Phelan-McDermid syndrome [66]. SHANK3 belongs to a family of adaptor proteins which interact with the actin cytoskeleton and different components of the postsynaptic density, and thus plays an important role in synapse development and function [67]. *SHANK3* KO in PSC-derived neurons results in decreased neuronal soma size, reduced neurite length and less neurite branching [68, 69], and a recent publication focusing on patients with de novo mutations in *SHANK3* reports decreased dendritic spine densities and volumes in *SHANK3*-mutated neurons [70]. In another study, placodal but not cortical neurons differentiated from iPSCs of ASD patients carrying microdeletions in the *SHANK3* locus and from *SHANK3* KO ESCs were found to have smaller cell somata and fewer synapses compared to control neurons, too, but in this study more extensive neurite branching has been observed [71]. Focusing on functional alterations, Thomas Südhof’s group observed an increased input resistance leading to augmented excitability in neurons derived from ESCs with conditional homozygous or heterozygous *SHANK3* loss-of-function mutations. In their elegant study, the authors revealed that the hyperexcitability of *SHANK3*-deficient human neurons is caused by a significant impairment of hyperpolarization-activated cation (I_h_) channels, which was accompanied by a decreased frequency and amplitude of EPSCs. Their work suggests that *SHANK3* interacts with hyperpolarization-activated cyclic nucleotide-gated (HCN) proteins, which generate I_h_ channels. *SHANK3* inactivation might thus contribute to the ASD pathogenesis via induction of an I_h_ channelopathy [69]. Notably, forebrain excitatory neurons derived from Phelan-McDermid syndrome patient iPSCs, which carry heterozygous 22q13.3 deletions comprising the *SHANK3* gene, were shown to exhibit an increased input resistance and decreased EPSC frequency and amplitude, too. These phenotypic changes selectively affected excitatory transmission, as no alterations in the amplitude and frequency of inhibitory postsynaptic currents were found. In this model, IGF1 treatment was demonstrated to increase the number of synapses that lacked SHANK3 but contained PSD95 and NMDARs with fast deactivation kinetics. Since such synapses showed rapidly decaying EPSCs—similar to synapses that appear in later stages of neurodevelopment [72]—the authors hypothesized that IGF1 rescues synaptic activity in Phelan-McDermid syndrome patient-derived neurons by promoting synapse maturation [73].

Finally, decreased enzymatic activity of the histone methyltransferase EHMT1, which is affected by heterozygous loss-of-function mutations associated to the ASD-related Kleefstra syndrome, was reported to result in decreased H3K9me2 levels at the promoter of the *GRIN1* gene, which encodes the NMDAR subunit 1. The consequently increased NMDAR/AMPAR ratio caused significant aberrations in neuronal network activity of *EHMT1* haploinsufficient neurons, which exhibited less regular network bursting as well as an overall decrease in burst frequency and an increase in burst duration. Inhibition of NMDARs by D-AP5 transiently ameliorated the NMDAR/AMPAR imbalance by driving the incorporation of AMPARs, and chronic treatment of EHMT1-deficient neurons with the NMDAR channel pore blocker MK-801 for 7 days even partially normalized network activity [74].

### Functional phenotypes in stem cell models of non-syndromic and idiopathic ASD

Interestingly, iPSC-derived neurons from patients with *SHANK2* haploinsufficiency, another member of the family of *SHANK* genes involved in ASD [75], displayed dendrite abnormalities and increased synapse numbers as well as a higher spontaneous EPSC frequency leading to a hyperconnected phenotype of *SHANK2*-deficient neurons [76]. Mutations in *NLGN4*, which encodes for a neuronal cell surface protein, too, might result in more active neuronal networks, since an increased number of excitatory synapses and higher frequency of miniature EPSCs have been reported for neurons differentiated from ESCs engineered to carry the ASD-associated R704C mutation in *NLGN4* [77]. Different results have been communicated regarding the functional consequences of mutations in *NRXN1*, which encodes for a presynaptic membrane cell adhesion protein. Avazzadeh et al. demonstrated that glutamatergic cortical neurons derived from *NRXN1α*^+/−^ iPSC lines exhibit increased frequency, duration and amplitude of calcium transients in comparison to control neurons derived from healthy donors [78]. On the other hand, neurons differentiated from patient iPSCs with biallelic *NRXN1α* deletion displayed a decreased amplitude and slower rise time of depolarization-evoked calcium transients, as well as a reduction in action potential amplitude [79]. In line with this, an earlier study by Pak et al. reported that glutamatergic neurons differentiated from engineered ESCs carrying heterozygous loss-of-function mutations in *NRXN1* show lower frequencies of miniature EPSCs compared to isogenic controls [80], and Kristen Brennand’s group, too, observed reduced neuronal network activity of glutamatergic iPSC-derived neurons with heterozygous intragenic *NRXN1* deletions. Specifically, the authors assessed two rare deletions in the 3′ region of the *NRXN1* locus and found that they affect NRXN1 splicing, resulting in the expression of mutant *NRXN1α* isoforms, which were not identified in neurons derived from either healthy controls or patients carrying deletions in the 5′ region of *NRXN1*. Interestingly, overexpression of 4 different wild-type *NRXN1α* isoforms could rescue the reduced neuronal network activity observed in 5′-NRXN1^+/−^ neurons, whereas it did not functionally impact 3′-NRXN1^+/−^ neurons. Overexpression of two of the newly identified mutant isoforms decreased the activity of neurons derived from controls and 5′-NRXN1^+/−^ carriers. This genotype-dependent mode of action of *NRXN1α* isoforms suggests the existence of a dominant-negative effect for specific heterozygous *NRXN1* deletions in the pathogenesis of *NRXN1*-related psychiatric disorders including ASD. Lastly, single-cell RNA sequencing analysis further revealed that besides exhibiting synaptic dysfunction, patient iPSC-derived neurons were underrepresented in cell clusters consisting of mature neurons. This suggests that *NRXN1* mutations might impair neuronal maturation, too [81].

Notable observations have also been made in the context of aberrations affecting the 16p11.2 chromosomal region, for which deletions as well as duplications have been associated with ASD [82]. Although penetrance of these mutations can be highly variable [45], Deshpande et al. found that iPSC-derived cortical neurons from deletion carriers showed an increase in neuronal soma size and dendrite length, which were associated with decreased excitability. In contrast, neurons with 16p11.2 duplication showed opposite morphological alterations (i.e., reduced cell size and dendrite length). These observations might at least partially explain the macro- and microcephalic phenotypes observed in 16p11.2 deletion and duplication carriers, respectively. Interestingly, neurons from both genotypes exhibited a reduction in synaptic density, which would be in line with common behavioral symptoms observed in 16p11.2 CNV carriers [83].

In 2015, Griesi-Oliveira et al. identified the ion channel coding gene *TRPC6* as a new candidate involved in non-syndromic ASD. Comparing control iPSC-derived NPCs and neurons with those of one ASD-affected individual with *TRPC6* haploinsufficiency, the authors found reduced calcium influx in NPCs and impaired neuronal maturation, as indicated by shorter and less branched neurites, as well as a reduction in the density of dendritic spines and VGLUT1-positive synaptic puncta. The observed neuronal phenotypes could be partially reverted by overexpression of TRPC6 or treatment with the TRPC6 agonist hyperforin. Importantly, the authors further demonstrated that the RTS-associated MECP2 acts as a positive transcriptional regulator of TRPC6 expression, and IGF1—which is able to revert the decrease of glutamatergic synapses in MECP2-mutant RTS-derived neurons [53] and SHANK3-mutant Phelan-McDermid syndrome-derived neurons [73]—rescued glutamatergic synapse numbers in *TRPC6*-mutant neurons as well [84].

Functional impairments in excitatory neurotransmission, characterized by a decreased frequency of AMPAR-mediated miniature EPSCs and a reduced amplitude of ionic currents elicited by NMDARs, have also been reported for neurons differentiated from patient iPSCs carrying (micro)deletions in the Xp22.11 chromosomal region affecting gene loci encoding the lncRNA *PTCHD1-AS* [85].

Using NGN2-based forward programming into glutamatergic neurons and CRISPR/Cas9-based gene inactivation, Deneault et al. investigated the effect of 10 additional ASD-related genes (i.e., *AFF2/FMR2*, *ANOS1*, *ASTN2*, *ATRX*, *CACNA1C*, *CHD8*, *DLGAP2*, *KCNQ2*, *SCN2A* and *TENM1*) on neuronal function. They found that KO of either of the genes *AFF2*/*FMR2*, *ASTN2*, *ATRX*, *KCNQ2* and *SCN2A* significantly reduced spontaneous EPSC frequency in iPSC-derived excitatory neurons. An increased action potential threshold was observed in *ATRX-, CHD8-, SCN2A-* and *TENM1*-KO neurons; ATRX- and *SCN2A-*KO neurons additionally exhibited decreased capacitance. Interestingly, KO of *SCN2A* further led to a reduction in action potential amplitude, mean firing rate and network burst activity [49].

Some of these results fit well with data generated with iPSCs from idiopathic ASD patients. For example, Liu et al. generated iPSC-derived neurons from 3 idiopathic male ASD patients and their unaffected male siblings, identifying in total 161 differentially expressed genes, of which 22 had previously been associated to ASD as for example *NRXN3* and *SCN2A* [86]. This and other studies on idiopathic ASD further revealed several functional alterations in iPSC-derived neurons including decreased calcium transients, decreased sodium and fast inactivating potassium voltage-gated currents, decreased spontaneous neuronal spiking and an earlier action potential saturation, as well as a reduced frequency and half width of spontaneous EPSCs [86, 87]. These findings are paralleled by data pointing to altered neurogenesis: In idiopathic ASD patients with early developmental brain overgrowth, increased proliferation of patient iPSC-derived NPCs positively correlated with the patients’ brain volumes and could be reverted by transfecting BRN2 or stabilizing β-catenin activity via lithium chloride treatment. In addition to altered proliferation, patient iPSC-derived neuronal precursors exhibited a differentiation bias towards inhibitory fates, resulting in an increased fraction of inhibitory neurons and reduced numbers of excitatory neurons with decreased glutamatergic synapse densities. Accordingly, patient-derived neurons produced less neuronal spikes and network bursts, which was successfully reverted by IGF1 treatment [88]. Analyzing the molecular causes of altered neurogenesis in this idiopathic ASD patient cohort in further depth, Schafer et al. assessed regulatory gene networks during neural induction and identified a gene module with different expression dynamics in patient iPSCs versus control cells. ASD neurons showed accelerated progression through this module, resulting in premature differentiation. They also provide evidence that these differences might be caused by increased chromatin accessibility in patient NPCs, leading to aberrant epigenetic imprinting. Interestingly, bypassing the proliferative NPC stage via an NGN2-based forward programming approach indeed normalized neurogenesis from ASD patient-derived iPSCs [89].

Taken together, data from a diverse collection of models reflecting syndromic as well as non-syndromic forms of ASD point to reduced complexity of neuronal morphology and functional alterations that might contribute to altered E/I balance, which is considered a key principle in the pathogenesis of ASD. Yet, these functional alterations are not uniform and encompass both hypo- and hyperactive changes. It remains to be determined how these diverse neuronal phenotypes might contribute to the emergence of the pathognomonic symptoms characteristic for this heterogenous neuropsychiatric disorder.

### Modelling the role of glia in ASD

Considering their important functions in the formation and maintenance of neuronal networks, it is conceivable that glial cells contribute to the pathogenesis of ASD. Indeed, iPSC-derived NPCs from an ASD patient with biallelic *NRXN1α* deletion proliferated slower and gave rise to an increased fraction of astrocytes upon differentiation [79], whereas shRNA-mediated knockdown of *NRXN1α* in PSC-derived NPCs led to impaired astrogenesis [90]. Differentiation of RTS patient iPSC-derived NPCs, too, resulted in a reduced number of astrocytes as compared to the differentiation of control iPSCs, and quantitative proteomic analysis revealed that the impaired astrogenesis was linked to an increased expression of the neurodevelopmental cell fate regulator LIN28 [91]. Williams et al. investigated the interaction between astrocytes and GABAergic interneurons in RTS and found that *MECP2*-mutant astrocytes had a detrimental effect on wild-type human interneurons, which showed a decrease in neurite length and terminal ends. Vice versa, wild-type astrocytes had a beneficial effect on patient interneurons resulting in increased neurite lengths and more terminal ends. Interestingly, treatment of mutant astrocyte-neuron co-cultures with either IGF1 or GPE—a peptide containing the first three amino acids of IGF1—positively influenced neuronal morphology, whereas it had negative effects on wild-type co-cultures. The authors hypothesize that decreased expression of IGF1 receptor in mutant astrocytes might contribute to the divergent responses observed after treatment [92]. Data from a study by Russo et al. further suggest that iPSC-derived astrocytes from non-syndromic ASD patients release increased amounts of reactive oxygen species and IL-6, which then impair neuronal morphology and synapse formation when co-cultured with control iPSC-derived neurons. Vice versa, control iPSC-derived astrocytes improved synaptogenesis of ASD-derived neurons, which were phenotypically characterized by fewer synapses, reduced glutamate release and a decrease in spontaneous neuronal activity. Treatment of mutant astrocytes with anti-IL-6 and control astrocytes with recombinant IL-6 mimicked the phenotype of the respective other condition [93]. Although studies on the role of non-neuronal cells in the pathogenesis of neuropsychiatric disorders are still at an early stage, these observations from stem cell models suggest that glial cells can significantly contribute to neuronal impairment in ASD.

### Non-genetic factors associated with ASD

In addition to inherited genetic pre-dispositions, non-genetic parental conditions might increase the offspring’s risk for developing a neuropsychiatric disease: Some groups reported that parental age might be positively correlated with the offspring’s ASD risk [94, 95]. In addition, maternal infections during pregnancy such as rubella or influenza have been linked to ASD [96–98]. Elevated levels of amniotic estrogens and prenatal exposure to progestin, too, have been reported to generate an increased risk of developing ASD during childhood [99]. The same applies to prolonged prenatal exposure to paracetamol [100–102], antidepressants [103] and valproic acid (VPA) [104]. While prescription of VPA during pregnancy is highly discouraged [105], it continues to serve as a proof-of-concept drug in stem cell-based models of developmental neurotoxicity testing (DNT) [106–108]. For example, Miranda et al. treated iPSCs with VPA during neural induction, simulating continuous in vivo exposure during the first 2–3 weeks of gestation. They observed disrupted neural rosette formation and decreased numbers of NCAM-positive cells [107]—neurogenic defects, which could, in principle, provide a basis for functional alterations.

Air pollutants such as nitrogen dioxide and particulate matter, too, have been associated with an increased risk of developing ASD [109–111]. Employing iPSC models, Yamada et al. studied exposure of neural cells to the environmental pollutant tributyltin [112] and the organophosphate and insecticide chlorpyrifos [113], both of which have been linked to an increased risk of ASD in offspring [114, 115]. Their data suggest that both substances lead to reduction of MFN1 and thus to mitochondrial dysfunction, which subsequently impairs neural induction [112, 113].

Childhood exposure to heavy metals such as mercury and lead is also considered a risk factor for developing ASD [116, 117]. Raciti et al. demonstrated that exposure of iPSC-derived neural cultures to subtoxic mercury concentrations promotes astrocyte differentiation [118], a phenotype reminiscent to the increased astrogliogenesis observed in ASD-related models of bi-allelic *NRXN1* deletions [79].

As in other fields, the use of higher throughput approaches has also been implemented in the context of DNT. Pei et al., for example, employed iPSCs and different iPSC-derived neural cell types such as NPCs, neurons and astrocytes to assess 80 different (developmental) neurotoxicants and environmental pollutants (including, e.g., chlorpyrifos, deltamethrin, diazepam, methyl mercuric (II) chloride, valinomycin and VPA), with 50 of them inducing cytotoxicity in at least one of the assessed cell types [119].

Although stem cell-based studies on DNT have, so far, mostly focused on alterations in neural induction and neurogenesis, it is conceivable that such changes might eventually elicit functional alterations. The fact that various environmental factors are discussed as contributors to ASD clearly warrants further work in this direction. As high throughput applications become increasingly available, such studies might, at some point, also accommodate DNT screens in genetic backgrounds predisposing to ASD.

## Prospects for studying ASD-related neuronal dysfunction in organoids

Classic 2D cultures are easy to grow and facilitate straightforward microscopic image analysis. However, they lack a number of aspects typical for tissues including proper formation of interstitial space and self-organization into organ-specific architectures such as for example the formation of cortical layers. Here, organoids have opened entirely new perspectives, especially for analyzing aspects of organogenesis and developmental defects over prolonged periods of time in vitro (Fig. 1; reviewed in detail by [120–122]). Up to now, however, only few studies have employed 3D systems to model functional alterations in ASD. Some of them relate to *DISC1* mutations, which have been identified as a high-risk genetic factor for a wide range of psychiatric disorders including SCZ and ASD [123]. Employing a multifaceted approach to investigate the molecular and cellular role of *DISC1* in the pathogenesis of psychiatric disorders, Ye et al. revealed that the C-terminal coiled-coil region of the DISC1 protein is crucial for its interaction with NDE1 and NDEL1. Mutations in *DISC1* were shown to result in a disruption of this interaction, leading to reduced NPC proliferation in ventricular zone-like structures of human forebrain organoids [124]. Concordantly, Srikanth et al. found impaired proliferation and structural abnormalities in human cerebral organoids derived from *DISC1*-mutant iPSCs, which could be rescued by WNT antagonism [125]. Although these organoid studies lack functional data, it is conceivable that reduced NPC proliferation at distinct phases of cortical development might lead to altered neuronal network formation.

Working on dorsal telencephalic organoids generated from idiopathic ASD patients with increased head circumference—a phenotype which has been associated with more severe autism symptoms [126–128]—Mariani et al. detected not only altered expression of genes related to cell proliferation, differentiation and synaptogenesis, but also an accelerated cell cycle and a *FOXG1*-dependent overproduction of GABAergic neurons [129].

An upregulation of *DLX* genes involved in GABAergic interneuron differentiation was also observed in telencephalic organoids with ASD-associated heterozygous loss of *CHD8.* Genes differentially expressed in *CHD8* mutant and wild-type organoids were further enriched for pathways relating to neurogenesis, neuronal differentiation, forebrain development, Wnt/β-catenin signaling and axon guidance [130]. Similar transcriptional changes were previously described for *CHD8*-mutant 2D cultures of NPCs and immature neurons [131]. Mechanistically, integration of RNA sequencing with genome-wide delineation of CHD8 binding in 2D cultures of iPSC-derived NPCs revealed that CHD8-bound genes were strongly associated with chromatin modification and transcriptional regulation, whereas indirectly downregulated genes (i.e., without CHD8-binding sites) were enriched for pathways involved in brain development, including synapse formation, neuron differentiation, cell adhesion and axon guidance [132].

A very recently published PSC-based KO model of the small GTPase RAB39B, which is associated with macrocephaly and ASD phenotypes, revealed increased proliferation and impaired differentiation of NPCs within human cortical organoids due to upregulated PI3K–AKT–mTOR signaling [133]. Larger ventricular zone-like structures as well as impaired neurogenesis have also been described in a 3D cortical model of syndromic RTS [134].

As for the functional consequences of altered neurogenesis, an ESC-based organoid model of Angelman syndrome revealed increased action potential frequency, as well as increased frequency and amplitude of calcium transients, reproducing the same phenotypes that were found in 2D neuronal cultures [64].

Newer models comprising fused organoids representing, for instance, dorsal and ventral forebrain, provide interesting prospects for studying the interplay between cortical excitatory and inhibitory neurons, in particular with respect to the migration and integration of ventrally born GABAergic interneurons into the dorsal cerebral cortex [135–137]. Previous studies in rodents had revealed that the L-type calcium channel KCC2 regulates interneuron migration by controlling intracellular calcium transients in response to GABA_A_ receptor activation [138]. Interestingly, the Timothy syndrome-associated L-type calcium channel Ca_v_1.2 might have similar implications for neuronal migration: Employing live imaging of fused brain spheroids, Sergiu Paşca and colleagues investigated the saltatory migration of GABAergic interneurons, born in ventral forebrain (subpallium) organoids, into dorsal forebrain (pallium) organoids. In this model, Timothy syndrome patient-derived GABAergic interneurons exhibited increased calcium signaling, resulting in an increased saltation frequency and concurrently decreased saltation length and speed, which could be rescued by pharmacologically blocking L-type calcium channels [135]. Again, it seems conceivable that such interneuron migration defects disrupt the tightly orchestrated liaison of inhibitory and excitatory neurons during cortex development and might thus elicit alterations in cortical function.

## Cell transplantation and disease modeling in vivo

Despite their numerous advantages in recapitulating early central nervous system (CNS) tissue formation, cerebral organoids lack a number of structures and cell types encountered in primary CNS tissue. Above all, the lack of a vascular system puts serious constraints on nutrient supply for long-term maintenance. Here, transplantation of human cell suspensions or even organoids into the rodent CNS might provide an avenue for extending stem cell-based disease modeling into an in vivo setting, thereby enabling the visualization of cell-autonomous and non-cell-autonomous pathophenotypes in a largely physiological though xenogeneic whole-brain context. Interesting data along this line have recently been reported in a study focusing on Down syndrome (DS). iPSC-derived NPCs from healthy controls and DS patients were transplanted into the brains of adult mice and traced with two-photon imaging. Neurons from both groups, controls and DS patients, exhibited axonal outgrowth, dendrite pruning and functional connections with host neurons, as indicated by the detection of miniature EPSCs, within an observation period of 4 months. In addition, neuronal bursts of defined spatiotemporal order and recurrent oscillatory behavior resembling neuronal activity in the human developing cortex could be recorded in these grafts. However, in vivo imaging revealed that in comparison to human control neurons, DS-derived neurons demonstrated higher dendritic spine stability marked by a decrease in dendritic spine turnover, which was accompanied by a reduction in network activity and graft-intrinsic oscillation [139]. As neural activity is an important regulator of synaptic plasticity, this study connects alterations in synaptogenesis with aberrant neural activity, which both seem to be at the core of pathogenic processes affecting early neurodevelopment in psychiatric disorders.

As a conceptual alternative to the transplantation of 2D cell culture-derived neural populations, Mansour et al. performed intracerebral transplantation of human brain organoids into the retrosplenial cortex of immunodeficient mice. Interestingly, the authors observed that the engrafted organoids developed long-distance axonal projections to many different brain regions including hippocampus, thalamus and hypothalamus and even underwent synaptic integration. The grafted tissue was also vascularized by host blood vessels, providing promising prospects for long-term in vivo studies [140]. In a slightly different scenario, Xu et al. dissociated control and DS patient-derived organoids into single cells and transplanted them into mouse brains, revealing that DS organoids generated an increased number of functionally active interneurons. Hypothesizing that the overproduction of inhibitory GABAergic neurons might be caused by OLIG2 upregulation, the authors performed shRNA-mediated knockdown of *OLIG2*, which resulted in normalization of interneuron differentiation [141] counteracting E/I imbalance.

Considering that dysregulated neuronal subtype generation and maturation might also be a pathomechanism relevant in ASD, these studies collectively suggest that transplantation of PSC-derived patient cells or organoids into an animal brain might be useful to decipher how different cellular processes conspire to elicit ASD phenotypes in a tissue context. Especially aspects such as cell migration, axon outgrowth and synaptic pruning might be more reliably mimicked in a tissue-like context, which provides a more physiological environment with blood vessels, immune cells and a pre-existing neuronal network. As of now, the accurate analysis of neuronal connectivity and network function in these complex systems still remains challenging. However, new functional imaging and reporter tools might open new avenues for assessing electrophysiological activity of both organoids and xenografts in much greater detail.

## Available methods for studying neuronal function and circuit formation in stem cell-based models

The currently available optical tools that enable monitoring of structure and functionality in stem cell-based in vitro models, as well as of grafted cells in vivo, can be divided into three main categories: (1) tools evaluating the structure of neurons and neural circuits, (2) tools reporting neuronal activity and (3) tools that allow the targeted modulation of this activity.

The first tool set mainly comprises virus-based tracer systems for the assessment of morphological and structural properties of neurons and neural circuits in stem cell-derived in vitro models and xenografts, which have already been used in animal models of ASD [20] and might prospectively be implemented in PSC-based disease models as well. In order to express different fluorescent proteins in the human cells of interest, mostly recombinant adeno-associated virus (rAAV)- or lentivirus (LV)-based tracer systems are utilized [142]. Since LVs, unlike rAAVs, integrate into the host cell genome and thus are passed onto the daughter cells after division, they can be efficiently used for transducing proliferative stem cell cultures for subsequent analysis of their neuronal progeny [143], whereas rAAVs are mainly employed to trace post-mitotic neurons [144–146]. Trans-synaptic tracers based on genetically modified recombinant rabies virus (RV) are particularly useful to study the connectivity of human stem cell-derived neurons both in vitro and in vivo after transplantation [145–148]. Using this system, we and others have demonstrated, for instance, that ESC-derived NPCs transplanted into the rodent hippocampus and striatum receive homotopic projections from the host brain [145, 146]. In a disease context, a RV-based trans-synaptic tracing study has revealed reduced connectivity in iPSC-derived neuronal cultures from patients with SCZ, which could be rescued by treating the SCZ iPSC-derived neurons with the antipsychotic drug loxapine [149]. It is to be expected that these tracing systems might be helpful to more comprehensively assess impaired synaptic connectivity in ASD as well.

The second set of optical tools enables the assessment of functional activity in neurons. The most prominent method for this purpose is calcium imaging, a method that measures variations in the intracellular calcium concentration as a proxy for neuronal activity, and calcium sensors such as Fluo-4AM were already successfully employed to study iPSC-based ASD models in 2D cultures [53] and 3D organoids [135]. These studies revealed a decreased frequency in calcium oscillations in RTS-derived neurons [53], whilst an increase in the overall intracellular calcium concentration was observed in neurons derived from patients with Timothy syndrome [135]. Genetically encoded calcium-indicators provide another route to assess functionality of grafted neurons. This was impressively illustrated by a recent study from Vanderhaeghen’s group, which demonstrated that grafted human ESC-derived cortical neurons become functional and undergo network integration in the mouse visual cortex [148]. Healthy iPSC-derived brain organoids grafted into the mouse brain are functionally active, too, as indicated by the occurrence of calcium oscillations in excitatory neurons within the transplanted organoid [140]. Voltage sensors responsive to changes in the cell membrane potential have been recombinantly expressed in healthy iPSC-derived neurons, too, and used to detect the occurrence of homeostatic plasticity of cell-intrinsic excitability—a very subtle form of synaptic plasticity, which had so far not been observed in human iPSC-derived neurons—that previously required laborious and time-consuming patch clamping [150]. Consequently, the use of such indicators might become especially attractive for potential future applications taking diseased iPSC-derived cells into a whole-brain context.

Lastly, the third set of optical tools comprising optically activated proteins, such as channelrhodopsin-II and halorhodopsins, allows to either excite (depolarize) or inhibit (hyperpolarize) neurons using blue or yellow light, respectively. These methods have been successfully used to study the effects of membrane potential changes in neurons evolving from mouse [151] and human ESCs *in vitr*o [140], as well as to control the activity of engrafted neurons [140, 143, 152]. In the context of a Parkinson’s disease animal model, for instance, transplantation of ESC-derived dopaminergic neurons ameliorates functional deficits, and light-induced suspension of graft activity abolishes this beneficial effect on amphetamine-induced rotation behavior [153]. Although these techniques have not yet been exploited in the context of ASD, they could potentially be used to selectively excite or inhibit neuronal subpopulations derived from patient PSCs, e.g., in order to decipher the relative contribution of neuronal subtypes to network activity and E/I (im)balance.

With the increasing move towards tissue-based models such as organoids and transplants, imaging technologies, too, need to be tailored towards more elaborate 3D analyses. Conventional sectioning and post-hoc immunohistochemistry of organoids or brains results in comparably limited 3D representation. Here, whole mount imaging techniques such as tissue clearing in conjunction with high-resolution 3D light sheet fluorescence imaging provide fast and reliable alternatives. This approach was, for instance, successfully used for assessing axonal projections of small molecule-induced cortical neurons [154] and RV-based trans-synaptic tracing of host–graft innervation [146]. Notably, light sheet fluorescence imaging of cleared tissue can also be combined with novel tissue expansion protocols allowing the depiction of neuronal circuits and synaptic connections in super-resolution [155].

In addition to optical tools and classical electrophysiological methods such as patch clamping, multi-electrode arrays (MEAs) have been used successfully to assess neuronal activity of stem cell-based systems. This technology enables functional recordings from PSC-derived neurons on a population level, including the assessment of network-related parameters such as network burst activity and synchrony. Notably, this technology is sensitive enough to detect subtle alterations in network activity such as, for instance, decreased synchronized bursting after inactivation of L-type calcium channels [156], which are implicated in neuropsychiatric disorders such as ASD [65]. The parameters assessed by MEAs are likely to be affected in the context of an E/I imbalance or other functional alterations discussed in the context of ASD, as was already demonstrated in an iPSC-based 2D model of idiopathic ASD [88]. While Trujillo et al. recently reported that MEAs can be used to detect oscillatory waves and neuronal network activity in long-term cultured healthy brain organoids [157], future studies might also exploit this technology for organoid-based disease modeling.

## Next generation tools for assessing neuronal connectivity and function in vivo

During the last couple of years, a vast array of additional high-end tools for assessing neuronal function and connectivity have been established in rodent systems. Translated to human stem cell-derived neurons, these tools could provide unprecedented insights into functional alterations associated with ASD and other neuropsychiatric diseases. As these read-out systems are streamlined towards application in the rodent brain, they might be particularly useful for studying altered connectivity and function of human neurons grafted into a rodent brain (Box 1).

**Box 1:**
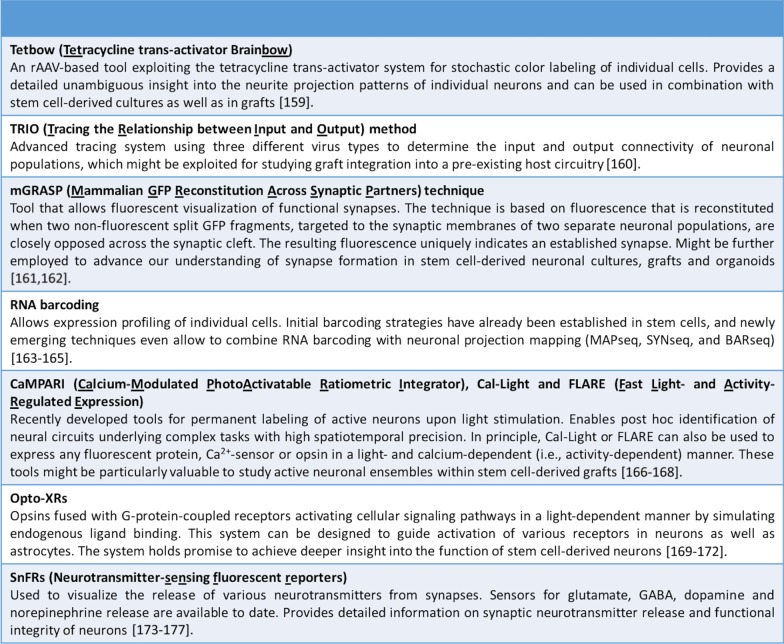
Next generation tools for assessing neuronal connectivity and function in vivo

Selected examples of novel tools originally developed for assessing connectivity and neuronal function in the rodent brain might lend themselves to the analysis of patient iPSC-derived neurons in 3D cultures and xenografts. Tools developed to study anatomical features include the Brainbow technology [158] and the novel Tetbow system recently employed in the context of rAAV-based labeling studies [159]. Both approaches enable labeling of multiple cells with individual colors and can thus provide a detailed unambiguous insight into neurite projection patterns.

Besides the mere detection and visualization of synaptically connected cells using well-established recombinant RV-based systems, the TRIO technology could serve as a valuable tool for assessing graft integration into the host circuitry. TRIO uses three different virus types to determine the input and output connectivity of diverse neuronal populations; the most recent cTRIO variant even confers cell-type specificity [160].

While these methods label synaptically connected neurons as such, the mGRASP system can be used to visualize individual synapses engaged in neuronal communication. The technology is based on functional complementation between two non-fluorescent GFP fragments across tightly juxtapositioned pre- and postsynaptic membranes [161] and can even be used in combination with optogenetic tools [162].

Novel techniques such as MAPseq, SYNseq and BARseq combine RNA barcoding and projection mapping, thereby enabling transcriptome analyses in connected cells [163–165]—certainly an interesting perspective for the study of graft-host interactions.

Another toolset comprises methods for the evaluation of a neurons’ activity state as well as detailed information on synaptic neurotransmitter release. Here, deeper insight into the actual function of stem cell-derived neurons could be achieved with advanced optical activity sensors and modulators. Currently applied conventional calcium sensors visualize neuronal activity in real time, posing limitations for post hoc analysis though. Since the fluorescence signal is lost after fixation, it is impossible to retrospectively determine, for instance, the cellular identity of the cells that have been engaged in neuronal activity within a piece of brain tissue. The recently developed activity sensor CaMPARI overcomes this limitation via a permanent green-to-red fluorescence conversion upon blue light stimulation [166]. As a result, CaMPARI allows for post hoc identification of active neurons and might thus be used in the context of stem cell grafts to permanently and selectively label active neurons at a specific point of time, which would allow an accurate evaluation of the number and type of neurons engaged in activity within a xenograft. Optical switch systems such as Cal-Light or FLARE operate along the same line and can, in principle, be used to express any fluorescent protein, calcium sensor or opsin in a light- and calcium-dependent manner exclusively in active neurons. This is not only attractive for studying neuronal dynamics within human transplants, but also for modulating the activity of grafted cells [167, 168].

Other variants for advanced optical activity modulation include Opto-XRs, which are opsins fused with G-protein coupled receptors, thus activating cellular signaling pathways in a light-dependent manner by simulating endogenous ligand binding. In vivo, Opto-XRs have been successfully used to study the role of different receptors in regulating complex behavior [169–172].

Finally, and a level deeper in complexity, neurotransmitter-sensing fluorescent reporters (SnFRs) even enable visualization of transmitter release from neuronal synapses and astrocytes; a number of SnFRs detecting synaptic glutamate, GABA, dopamine and norepinephrine release have already been established [173–177].

Despite the reasonable excitement accompanying these technological advances, implementation of such complex read-outs for studying disease-related alterations in human 3D cultures and xenografted human neurons will be challenging. Donor cell preparations will have to be highly defined and homogenous to minimize intergraft variability. Increased heterogeneity, as currently experienced, for example, in many organoid preparations and grafts, could result in highly variable and hardly interpretable connectivity patterns. For example, surgical variability in graft placement, differing degrees of cell survival, heterogeneity with respect to in situ cell maturation and immunological factors could significantly interfere with the functional analysis of human neurons in 3D cultures and xenografts.

## Stem cell-based models for neuropsychiatric drug development

Besides increasing our understanding of the pathophysiological mechanisms underlying neuropsychiatric diseases, PSC-based models represent valuable platforms for the identification and validation of candidate pharmacological compounds for the treatment of these disorders. Kumari et al. [178] and Kaufmann et al. [179], for instance, screened thousands of compounds for their ability to increase FMR1 expression. Although the authors only identified a small number of compounds exhibiting modest efficacy, including an HDAC inhibitor [179], these studies illustrate the feasibility of using PSC-derived disease models for drug screening. More recently, Vershkov et al. used FXS iPSCs to test a library of 140 compounds for their ability to rescue the expression of *FMR1*. A combination of the DNA methyltransferase inhibitor 5-azadC and the SAH hydrolase and histone methylation inhibitor DZNep was most effective in iPSCs, whereas a sustained increase in *FMR1* expression in FXS iPSC-derived NPCs was even observed after treatment with 5-azadC alone. Importantly, such treatments were also effective in vivo, since treatment with 5-azadC and DZNep or 5-azadC alone after transplantation of FXS iPSCs or FXS iPSC-derived NPCs into mouse brains, respectively, resulted in a sustained increase in *FMR1* expression [180]. Along a similar line, Darville et al. screened more than 200 compounds for their capability to increase *SHANK3* expression in iPSC-derived neurons from patients with *SHANK3* haploinsufficiency and identified lithium and VPA as most effective candidates in this regard. These two compounds even increased the number of SHANK3-containing synapses and successfully reverted the decreased intensities and frequencies of spontaneous calcium oscillations associated with *SHANK3* haploinsufficiency. As a proof-of-concept for the validity of their screening results, the authors administered lithium to an ASD patient, whose iPSC-derived neurons exhibited increased SHANK3 expression upon lithium exposure in vitro. After 1 year of lithium administration, this patient indeed showed improved social behavior and cognitive performance [181]. These preliminary findings point to interesting prospects of the iPSC technology for personalized medicine, as they indicate that patient-specific iPSC-derived neurons could, in principle, be employed as a platform to screen for effective compounds in an individualized manner.

## Future perspectives and concluding remarks

Although highly defined PSC-derived neuronal cultures facilitate the study of cell type-specific alterations relevant for psychiatric disorders, they do not represent physiological neuronal circuitries, which are composed of a variety of different neuronal as well as glial cell types. On the other hand, reductionist approaches may come with their own advantages. For example, for assessing E/I balance—a phenomenon discussed as relevant for a number of psychiatric diseases—highly defined populations of excitatory and inhibitory neurons generated by forward programming could be very helpful. Stoichiometrically mixed and equipped with distinct fluorescent reporter genes, such co-culture systems could reveal how each individual cell type contributes to altered network formation and activity in ASD. More complex disease models based on the co-culture of neuronal and non-neuronal cells such as astrocytes, oligodendrocytes and microglia might be informative for investigating non-cell-autonomous effects relevant for disease development. Whilst such advanced 2D culture systems might be well suited for high-throughput genetic and compound screens, 3D settings such as organoids or neurotransplantation could provide a tissue-like 3D environment and thus increase the authenticity of human neuronal model systems. In contrast to classic animal models, xenografts provide the possibility to investigate human-specific gene variants in human cells in vivo. This can provide an advantage over mouse models because many disease-related genes do not have mouse orthologs and human cells might respond different to pathogenic stimuli than mouse cells [182]. Prospectively, xenograft approaches could also open an avenue to assess the effect of compounds on disease-relevant human cells in a living mammalian brain.

In conclusion, PSC-based 2D and 3D models represent powerful platforms to (1) investigate the pathophysiological mechanisms underlying genetically defined and potentially idiopathic forms of ASD, (2) test how environmental factors such as prenatal exposure to certain chemicals and drugs predisposes to ASD and (3) identify new treatment strategies for this neuropsychiatric disease. While there is a need to further improve the generation of highly standardized cell systems, the combination of iPSC technology, genome editing and novel functional read-out systems has the makings to transform our capabilities for studying the pathogenesis and treatment of neuronal dysfunction in ASD and other neuropsychiatric disorders.

## Data Availability

Not applicable.

## Abbreviations

AAVS1: Adeno-associated virus site 1
AFF2: AF4/FMR2 family member 2
AKT (aka PKB): Protein kinase B
AMPAR: α-Amino-3-hydroxy-5-methyl-4-isoxazolepropionic acid receptor
ANOS1: Anosmin 1
ASCL1: Achaete–scute family bHLH transcription factor 1
ASD: Autism spectrum disorder
ASTN2: Astrotactin 2
ATRX: ATRX chromatin remodeler
5-azadC: 5′-Aza-2′-deoxycytidine
BK channels: Big potassium channels
BRN2 (aka POU3F2): Brain-2
CACNA1C: Calcium voltage-gated channel subunit alpha 1 C
CDKL5: Cyclin-dependent kinase-like 5
CHD8: Chromodomain helicase DNA-binding protein 8
CLCN3: Chloride voltage-gated channel 3
CNS: Central nervous system
CNV: Copy number variation
CREB: CAMP (Cyclic adenosine monophosphate) response element-binding protein
CRISPR/Cas9: Clustered regularly interspaced short palindromic repeats (CRISPR)-associated protein 9
CTIP2: COUP (chicken ovalbumin upstream promoter)-transcription factor-interacting protein 2
DCC: DCC netrin 1 receptor
DISC1: Disrupted in schizophrenia 1
DLGAP2: DLG-associated protein 2
DLX: Distal-less homeobox
DNT: Developmental neurotoxicity
DS: Down syndrome
DZNep: 3-Deazaneplanocin A
EHMT1: Euchromatin histone lysine methyltransferase 1
E/I: Excitation–inhibition
EPSC: Excitatory postsynaptic current
ESC: Embryonic stem cell
FMR1: FMRP translational regulator 1
FMRP: Fragile X mental retardation 1 protein
FOXG1: Forkhead box G1
FXS: Fragile X syndrome
FURIN: Coding for furin, paired basic amino acid cleaving enzyme
GABA: Gamma aminobutyric acid
GRIA: Glutamate ionotropic receptor AMPA-type subunit
GRIN: Glutamate ionotropic receptor NMDA-type subunit
GRM: Glutamate metabotropic receptor
IGF1: Insulin-like growth factor 1
IL-6: Interleukin 6
iPSC: Induced pluripotent stem cell
KCC2 (aka SLC12A5): Potassium chloride transporter member 5
KCNQ2: Potassium voltage-gated channel subfamily Q member
KO: Knockout
LV: Lentivirus
MEAs: Multi-electrode arrays
MECP2: Methyl-CpG-binding protein 2
MEK/ERK: Mitogen-activated protein kinase (MAPK)/extracellular signal-regulated kinase (Erk) kinase
MFN1: Mitofusin 1
mGluR5: Metabotropic glutamate receptor 5
MRI: Magnetic resonance imaging
mTOR: Mechanistic target of rapamycin
NCAM: Neural cell adhesion molecule
NDE1: NudE neurodevelopment protein 1
NDEL1: NudE neurodevelopment protein 1-like 1
NGN2: Neurogenin 2
NLGN3/4: Neuroligin 3/4
NMDA: *N*-Methyl-d-aspartic acid
NPC: Neural progenitor cell
NRXN1: Neurexin 1
OTX2: Orthodenticle homeobox 2
PAX6: Paired box 6
PI3K: Phosphoinositide 3 kinase
PSC: Pluripotent stem cell
PSD95: Postsynaptic density protein 95
PTCHD1-AS: Patched domain-containing protein 1-antisense RNA
rAAV: Recombinant adeno-associated virus
RAB39B: Ras-related protein Rab-39B
REST: RE1 silencing transcription factor
ROBO3: Roundabout guidance receptor 3
RTS: Rett syndrome
RV: Rabies virus
SAH: *S*-Adenosylhomocysteine
SCN2A: Sodium voltage-gated channel alpha subunit 2
SCZ: Schizophrenia
SHANK2/3: SH3 and multiple ankyrin repeat domains 2/3
SHH: Sonic hedgehog signaling molecule
SLIT1: Slit guidance ligand 1
SMAD: Small mothers against decapentaplegic
SNAP91: Synaptosome-associated protein 91
SNP: Single nucleotide polymorphism
SOX1: SRY (sex-determining region Y)-box transcription factor 1
TBR1: T-box brain transcription factor 1
TENM1: Teneurin transmembrane protein 1
TRPC6: Short transient receptor potential channel 6
TSNARE1: T-SNARE domain-containing 1
UBE3A: Ubiquitin protein ligase E3A
VGLUT: Vesicular glutamate transporter
VPA: Valporic acid
WNT: Wingless and int-1
